# Eculizumab in the Treatment of Aquaporin-4 Seronegative Neuromyelitis Optica Spectrum Disorder: A Case Report

**DOI:** 10.3389/fneur.2021.660741

**Published:** 2021-05-06

**Authors:** Lakshmi Digala, Nakul Katyal, Naureen Narula, Raghav Govindarajan

**Affiliations:** ^1^Department of Neurology, University of Missouri Health Care, Columbia, MO, United States; ^2^Department of Pulmonary and Critical Care, Northwell Health – Staten Island University Hospital, New York, NY, United States

**Keywords:** autoimmune disease, neuromyelitis optica spectrum disorder, seronegative, eculizumab, aquaporin-4, complement-inactivating agents, relapses

## Abstract

**Objective:** To report the case of a 35-year-old woman with treatment-resistant aquaporin-4 (AQP-4) immunoglobulin G (IgG) seronegative neuromyelitis optica spectrum disorder (NMOSD) successfully treated with eculizumab (a terminal complement inhibitor).

**Methods:** The investigational procedures and treatment regimens the patient received were documented over 8 years [2012 (first presentation) to 2020].

**Results:** The patient presented with subacute onset of lower-limb weakness and numbness, gait imbalance, and urinary incontinence. Magnetic resonance imaging (MRI) showed abnormalities in the thoracic spine from T7 to T10, but brain and cervical spine scans, visual evoked potential latencies, and IgG index were normal; cerebrospinal fluid pleocytosis and oligoclonal bands were both present. After treatment with intravenous methylprednisolone 1 g/day for 5 days, the patient was discharged without medication to acute rehabilitation but experienced relapses from 2012 to 2014. She was treated with oral prednisone (initiated at 40 mg/day in 2014; the dose was halved in 2015 due to weight gain) and mycophenolate mofetil (MMF) 1 g twice daily (from June 2015), but between 2014 and 2019 experienced 4–5 relapses/year, requiring treatment with intravenous methylprednisolone, with added maintenance plasma exchange from 2018 onwards. Although the patient tested negative for antibodies to AQP-4 and myelin oligodendrocyte glycoprotein, she was diagnosed with NMOSD in February 2017, based on recurrent episodes of longitudinal extensive transverse myelitis, MRI changes, and area postrema syndrome. By 2018 the patient needed a cane to walk. Prednisone and MMF were discontinued mid-2018, and rituximab was prescribed from July 2018 (maintenance regimen two 1 g doses 2 weeks apart every 6 months) but discontinued in July 2019 owing to lack of significant improvement. From July 2019 eculizumab was prescribed for 6 months (900 mg weekly for the first four doses, then 1200 mg every 2 weeks). The patient had no relapses or adverse events during and after eculizumab treatment (as of August 2020) and was able to walk unaided; her Expanded Disability Status Scale score improved from 4–5 during 2015–2018 to 2 in 2020 following eculizumab treatment.

**Conclusion:** Eculizumab shows promise as a treatment for AQP-4 IgG-seronegative NMOSD and further studies are warranted.

## Introduction

Neuromyelitis optica (NMO)/NMO spectrum disorder (NMOSD) is a rare disease characterized by autoimmune demyelination and axonal damage predominantly affecting the spinal cord [longitudinal extensive transverse myelitis (LETM)] and optic nerve (optic neuritis) (1). It is preponderant in women, and the median age of onset is 39 years (2). For many years, NMOSD was considered to be a variant of multiple sclerosis, but in 2004 the identification of aquaporin-4 (AQP-4) immunoglobulin G (IgG) antibodies in a subset of patients led to the differentiation of NMOSD from multiple sclerosis (3, 4). The anti-AQP-4 antibody selectively binds the AQP-4 water-channel protein in astrocytes and is detected in up to 73% of patients with NMOSD (5). AQP-4 antibodies activate the complement cascade, leading to the breakdown of complement protein 5 (C5) to C5a and C5b, and generation of the membrane attack complex (MAC; C5b-9) (6). Of the remaining patients with NMOSD, 11% are positive for antibodies to myelin oligodendrocyte glycoprotein (MOG)—a component of myelin expressed on the surface of myelin sheaths in the central nervous system (CNS) (7)—and 16% have no detectable antibodies to either AQP-4 or MOG (4, 5).

NMOSD is characterized by unpredictable recurrent episodes of optic neuritis and myelitis. Partial recovery occurs between attacks, but neurologic disability accumulates, including blindness and paralysis (8). The conventional treatment of an acute episode of seronegative NMOSD is intravenous corticosteroids, with or without plasma exchange (PLEX). Various immunosuppressive therapeutic strategies, such as mycophenolate mofetil, azathioprine, and rituximab, are useful in the prevention of relapses.

Eculizumab is a recombinant humanized monoclonal antibody that binds to complement component C5 and prevents its conversion to C5a and C5b (9). The precise mechanism by which eculizumab exerts its therapeutic effect in NMOSD is unknown, but it is presumed to involve inhibition of AQP-4-antibody-induced MAC generation and associated astrocyte loss (9). It was approved in 2019 by the US Food and Drug Administration as the first drug for the treatment of patients with NMOSD who are anti-AQP-4 antibody positive (10). Here we report a case of a woman diagnosed with treatment-resistant AQP-4 IgG-seronegative NMOSD, who responded well to eculizumab without any adverse effects. To our knowledge, this is the first such case reported in the literature.

## Case Report

We describe the case of a 35-year-old white woman. She initially presented in July 2012 at the age of 27 years, when she was working as a nurse. She reported subacute onset of lower limb weakness (strength 3/5 on the Medical Research Council scale), lower limb numbness, gait imbalance, and urinary incontinence. Magnetic resonance imaging (MRI) of the thoracic spine, with and without contrast, demonstrated signal abnormality from T7 to T10 associated with post-contrast enhancement. MRI of the brain and cervical spine, with and without contrast, was normal. Visual evoked potential (pattern VEP) showed normal P100 latencies. A lumbar puncture showed cerebrospinal fluid pleocytosis (white blood cells: 20 cells/dL, with predominant lymphocytes), normal IgG index, and oligoclonal bands.

**Figure 1:**
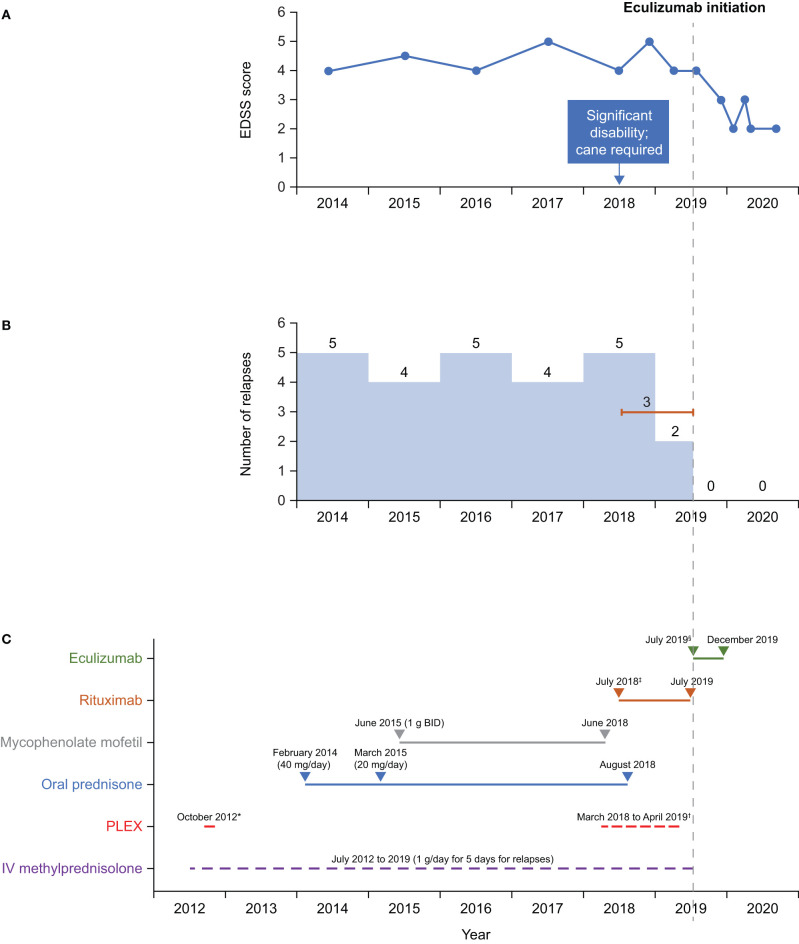
Timeline of **(A)** EDSS scores, **(B)** relapses, and **(C)** treatment. *Rituximab dose: 2 × 1 g, 2 weeks apart, then every 6 months. ^†^Eculizumab dose: 900 mg weekly (first four doses) then 1,200 mg every 2 weeks. ^‡^5 exchanges on alternate days. ^§^3–5 exchanges on alternate days every 4 weeks. BID, twice daily; EDSS, Expanded Disability Status Scale; IV, intravenous; PLEX, plasma exchange.

**Figure 2:**
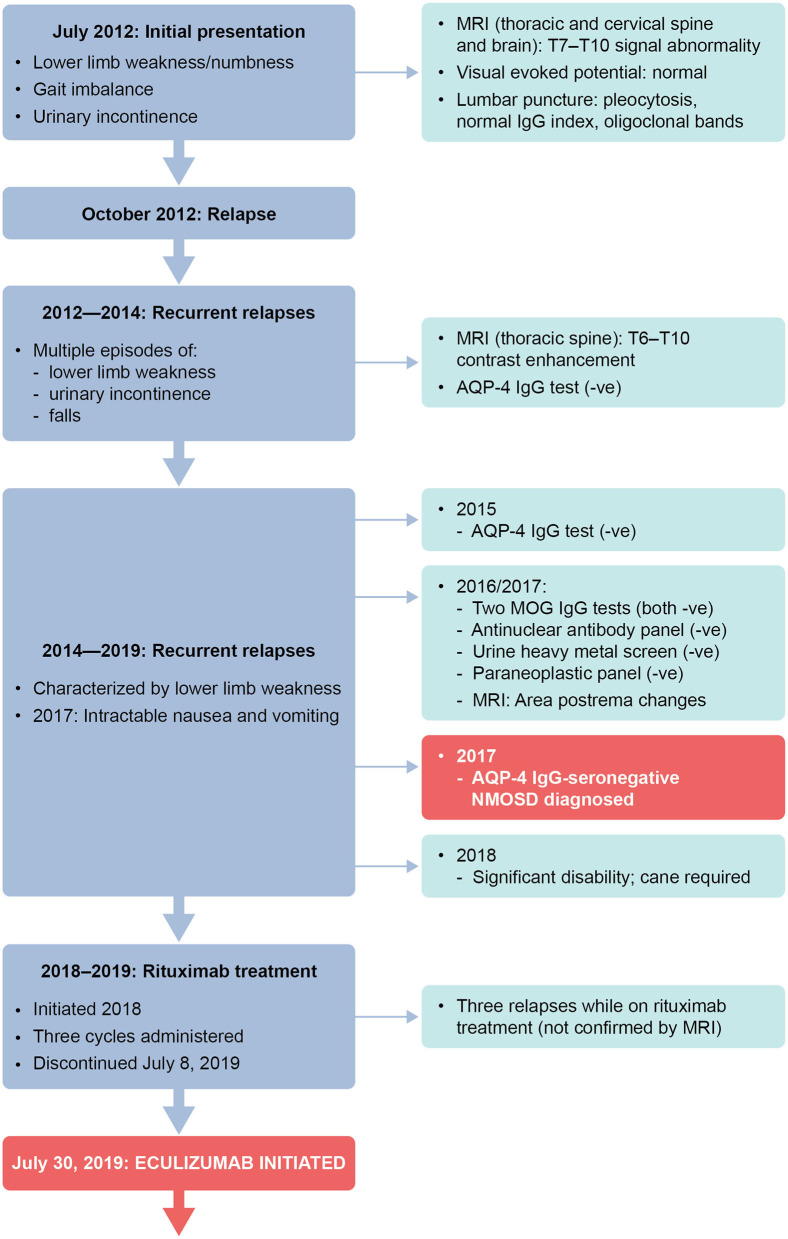
Timeline of clinical presentation and investigations. –ve, negative; AQP-4, aquaporin-4; IgG, immunoglobulin G; MOG, myelin oligodendrocyte glycoprotein; MRI, magnetic resonance imaging.

A summary of treatment regimens and timelines is shown in Figure 1 and the clinical course is described in Figure 2. Following her initial presentation the patient was admitted to hospital and treated with intravenous methylprednisolone at a dose of 1 g/day for 5 days, after which she was discharged without medication to acute rehabilitation. She had a relapse in October 2012 and underwent PLEX every other day for a total of five exchanges which resolved the episode; a relapse was defined as patient-reported symptoms or any new signs consistent with CNS lesions and attributable objective changes in MRI or visual evoked potential. The patient was treated with intravenous methylprednisolone for relapses between 2012 and 2014, during which she experienced multiple episodes of lower limb weakness, urinary incontinence, and falls. During these relapses, MRI of the thoracic spine showed contrast enhancement from T6 to T10. In 2014, the patient tested negative for AQP-4 IgG and in February she was initiated on oral prednisone 40 mg/day. Her Expanded Disability Status Scale (EDSS) score was 4 at that time.

In 2015, testing for anti-AQP-4 antibodies by fluorescence-activated cell sorting (FACS) proved negative. In March of that year, the patient's oral prednisone dose was reduced to 20 mg/day because of weight gain. In June 2015, the patient was initiated on mycophenolate mofetil 1 g twice daily.

In 2016 and 2017, testing for MOG IgG (FACS assay) was negative; tests for antinuclear antibody panel, urine heavy metal screen, and paraneoplastic panel were also negative. In February 2017, following episodes of intractable nausea and vomiting with associated area postrema changes on MRI, the patient received a diagnosis of seronegative NMOSD from a National Multiple Sclerosis Society Center of Excellence, based on recurrent episodes of LETM, MRI changes, area postrema syndrome, and lack of anti-AQP-4 antibodies. The patient continued to experience relapses between 2014 and 2019, with 4–5 relapses per year (Figure 1), characterized by lower limb weakness. On average, the patient was hospitalized three times a year for treatment of relapses with intravenous methylprednisolone 1 g/day for 5 days, with maintenance PLEX applied every 4 weeks from March 2018 to April 2019 as a total of 3–5 exchanges on alternate days. In June 2018, the patient stopped mycophenolate mofetil treatment as she felt it was not effective. Between 2014 and 2018, the patient's EDSS score was 4–5, and she eventually needed a cane to walk in 2018.

Starting in July 2018, rituximab 375 mg/m^2^ was administered intravenously every week for 4 weeks, then as two 1 g doses, 2 weeks apart every 6 months. Rituximab has not been approved for treatment of NMOSD; this regimen is the same as that used in a Phase 3 trial in patients with NMOSD (11). In August 2018, the patient stopped oral prednisone treatment [due to weight gain of 20 lb (~9 kg) in 2 months, acne, and mood issues]. Rituximab treatment was stopped after the second maintenance cycle was completed on July 8, 2019, as no significant improvement in relapses occurred—the patient experienced three relapses while receiving rituximab (not confirmed by MRI). CD19^+^ B cells were depleted during rituximab therapy, with the median cell count for the last 6 months of treatment being 0.001 × 10^9^cells/L (range 0–0.0159 × 10^9^ cells/L). Eculizumab infusion was initiated on July 30, 2019, with the approval of the patient's insurance provider following a peer-to-peer review by one of her physicians (RG) and the provider. Two weeks before eculizumab initiation, the patient was vaccinated against *Neisseria meningitidis* with meningitis ACWY and B vaccines, according to the recommendations of the Centers for Disease Control and Prevention's Advisory Committee on Immunization Practices (12). She then received the recommended dose of eculizumab 900 mg weekly for the first four doses, followed by 1,200 mg every 2 weeks starting 4 weeks after initiation. While treated with eculizumab, the patient showed improvements on the EDSS (score of 2–3; lower scores indicate less disability) (Figure 1) and she experienced no relapses or adverse events. Eculizumab was discontinued in December 2019 when the patient's insurance provider denied continued coverage despite peer-to-peer review. At subsequent follow-up visits after eculizumab discontinuation and as of August 2020 she has remained relapse-free and symptom-free and is not taking any medication for NMOSD. The patient can walk without any aids, has an EDSS score of 2, and works full time as a physician's assistant.

## Discussion

The underlying cause of NMOSD is primarily humoral-mediated autoimmunity, resulting in florid demyelination and inflammation (8). Although detection of anti-AQP-4 antibodies is a critical diagnostic step in diagnosing NMOSD, a more challenging testing sequence is necessary for diagnosing NMOSD in seronegative patients in order to exclude a variety of diseases mimicking NMOSD (13).

The core clinical characteristics of NMOSD constitute acute myelitis, optic neuritis, area postrema syndrome, acute brainstem syndrome, symptomatic cerebral syndrome with NMOSD-typical brain lesions, and symptomatic narcolepsy or acute diencephalic clinical syndrome with typical NMOSD-diencephalic MRI lesions (2). To meet the criteria for the diagnosis of seronegative NMOSD, patients must have experienced at least two core characteristics and at least one of the three most common characteristics (optic neuritis, acute myelitis with LETM, or area postrema syndrome with associated MRI lesions). This patient's core clinical characteristics were recurrent LETM, area postrema syndrome, and absence of AQP-4 antibody.

There are interesting differences between patients who are seropositive and seronegative for AQP-4 antibodies: the seronegative disease population does not show the female predominance of the seropositive patients, comprises a higher proportion of white people, and is associated with a younger mean age at onset (1, 13, 14). There are also differences in disease characteristics between the two groups. Although there are few differences in the time to relapse, annualized relapse rate, recovery from relapse, annualized EDSS increase, and mortality rate, seronegative patients are more likely to present with both optic neuritis and LETM than seropositive patients (14, 15).

The crucial role of the complement cascade in NMO pathogenesis is supported by the fact that NMO-like lesions were only reproducible in an animal model when human complement was co-administered (16). The pathophysiology associated with anti-MOG antibodies is less well-characterized, but they have also been shown to activate the complement cascade (15, 17). Similar to the patient described in this report, a small proportion of patients with NMOSD are seronegative for both antibodies, with no detectable AQP-4 IgG or MOG IgG. The pathophysiology in this patient population is poorly understood (18), although complement-mediated damage can be seen in both seropositive and seronegative cases (6, 19). Although our patient might be described as being “double seronegative”—as patients who are seronegative for both AQP-4 IgG and MOG IgG are sometimes referred to in the literature—there is ongoing debate about how this subgroup should be classified and further research is required (18).

In such patients, the presence of low antibody titers or autoantibodies against aquaporin-1 (AQP-1), another water channel in CNS astrocytes, has been suggested to be associated with the development of NMOSD (20, 21). Other data suggest that seroconversion to negative status may occur with immunotherapy and that patients should be retested for AQP-4 IgG during relapses and before immunotherapy (8). There is also the possibility that there is an as yet unidentified target for complement activation (6, 19).

It follows that inhibition of complement activation should reduce damage to astrocytes in patients with AQP-4-IgG-positive NMOSD. Eculizumab prevents the cleavage of C5 to C5a and C5b, and significantly reduced the relapse risk in patients with AQP-4-IgG-positive NMOSD compared with placebo in a large Phase 3 trial, leading to its approval for this condition (22).

Eculizumab is not approved for seronegative NMOSD. Given the pathological similarities between seropositive and seronegative NMOSD, we decided to try eculizumab in our patient. A distinct improvement was seen during the 6 months of eculizumab treatment. The last dose of rituximab was given 22 days before eculizumab initiation. Although there may have been some overlap in activity between the two drugs, we believe the subsequent improvement in the patient's condition to have been associated with eculizumab treatment, given that the patient experienced three relapses while receiving rituximab. Before eculizumab initiation, the patient's EDSS score was 4–5, but it reduced to 3 during eculizumab therapy, at which point the patient was also relapse-free (Figure 1). There is a common misconception that damage caused during NMOSD relapse is irreversible; however, the improvements in EDSS score seen in our patient are corroborated by the findings of several studies reporting reductions in EDSS scores in response to targeted treatment (23, 24). Our patient remained relapse-free during the 12 months after stopping eculizumab therapy, even though she received no other therapy.

Although the repeated testing suggests that this patient did not have anti-AQP-4 or anti-MOG antibodies, it is possible that she harbored a low titer that may have been detectable with a more sensitive assay. Alternatively, she might have complement-activating antibodies to AQP-1 or a currently unidentified target. Outside of the research setting it may be difficult to conclusively identify the pathology; however, our experience suggests that a trial of eculizumab may be warranted in patients with AQP-4 IgG-seronegative NMOSD.

## Conclusion

Eculizumab shows promise as a treatment for AQP-4/MOG IgG-seronegative NMOSD, and further studies are warranted to explore the possibility of using this treatment in patients with treatment-resistant seronegative NMOSD. The mechanism of action of eculizumab in seronegative NMOSD remains to be elucidated.

## Summary

Neuromyelitis optica spectrum disorder (NMOSD) is a rare autoimmune disease that affects the spinal cord and optic nerve, causing severe disability. Despite standard treatment, including off-label oral and intravenous corticosteroids and immunosuppressants, patients often continue to have recurrent attacks. Eculizumab has been approved for patients with NMOSD who have autoantibodies to the protein aquaporin-4 (AQP-4); eculizumab blocks the damage to nervous-system cells triggered by these antibodies. The patient described in this case report belongs to a minority of patients with NMOSD who are seronegative for anti-AQP-4 antibodies and for antibodies to another protein implicated in NMOSD—myelin oligodendrocyte glycoprotein (MOG). The woman presented in 2012 (then aged 27 years) and was diagnosed with AQP-4 IgG-seronegative NMOSD in 2017. She experienced frequent relapses between 2012 and 2019, despite receiving courses of standard treatment and rituximab, and eventually needed a cane to walk. After receiving eculizumab for 6 months in 2019, the patient experienced improvements in her level of disability and no longer experienced relapses. At follow-ups after treatment discontinuation, the patient continued to be relapse-free and could walk unaided. These findings suggest that eculizumab may be a useful addition to treatment options in the subgroup of patients with AQP-4-antibody seronegative NMOSD.

## Data Availability Statement

The original contributions presented in the study are included in the article, further inquiries can be directed to the corresponding author.

## Ethics Statement

Ethical review and approval was not required for the study on human participants in accordance with the local legislation and institutional requirements. The patient provided her written informed consent to participate in this study. Written informed consent was obtained from the individual for the publication of any potentially identifiable images or data included in this article.

## Author Contributions

All authors: conception, organization, execution of the research described in the manuscript, review, and critique of the manuscript.

## Conflict of Interest

RG has served on advisory committees for Alexion Pharmaceuticals, argenx, and Catalyst Pharmaceuticals, and is a member of the speakers' bureaux for Alexion Pharmaceuticals and Catalyst Pharmaceuticals. The remaining authors declare that the research was conducted in the absence of any commercial or financial relationships that could be construed as a potential conflict of interest.
